# *In vitro* Prebiotic Effects of Bamboo Shoots and Potato Peel Extracts on the Proliferation of Lactic Acid Bacteria Under Simulated GIT Conditions

**DOI:** 10.3389/fmicb.2018.02114

**Published:** 2018-09-05

**Authors:** Kiran Thakur, Guan-Yi Xu, Jian-Guo Zhang, Fang Zhang, Fei Hu, Zhao-Jun Wei

**Affiliations:** ^1^School of Food Science and Engineering, Hefei University of Technology, Hefei, China; ^2^Anhui Huaheng Biotechnology Co., Ltd., Hefei, China; ^3^Anhui Province Key Laboratory of Functional Compound Seasoning, Anhui Qiangwang Seasoning Food Co., Ltd., Jieshou, China

**Keywords:** lactic acid bacteria, prebiotic, potato peel, bamboo shoot, GIT

## Abstract

The present study explored the possible prebiotic application of potato peel and bamboo shoot extracts for the proliferation of lactic acid bacteria (LAB) from diverse niches and their tolerance ability to simulated gastrointestinal tract (GIT) conditions was also examined. Initially, the complete 16S rDNA sequencing of selected isolates revealed them as *Lactobacillus paracasei* (6), *Staphylococcus simulans* (2), and *Streptococcus thermophilus* (1). Higher cell densities and rapid pH change were obtained from cultured media supplemented with BS (2%) and PP (2%) as a carbon source. Their higher tolerance and the lowest reducing sugar abilities were obtained for BS at pH 2.5 and 9.0, while at pH 3.5 and 8.0 for PP. The isolates were screened for additional functional and technological properties to harvest the most appropriate starter. The selected isolates harbored promising functional properties such as amylase presence, cell surface hydrophobicity, autoaggregation, proteolytic and lipolytic activity, antifungal action, as well as exopolysaccharide production. On the basis of these attributes, microencapsulated strain K3 was found resistant to gastrointestinal conditions after 2 h, resulting in significantly (*p* ≤ 0.05) improved survival compared to non-capsulated strain. The current approach presents an interesting economical strategy to modulate LAB through supplementation of plant-derived carbon sources as well as to enhance their survival under GIT.

## Introduction

Lactic acid bacteria (LAB), known as the powerhouse of dairy industry with proven health benefits, are abundant in nature and they are mostly used in food and fermentation industry (Thakur et al., 2016b). The success of any food industry is generally centered on the cost of carbon substrates used during microbial fermentation to stimulate the growth of starter bacteria and accelerate the fermentation process. Due to the high demand for probiotic foods, which contain live bacteria, there is a continuous need to provide the novel and cost-effective sources of nutrition for these bacteria. There comes the role of prebiotic which constitutes non-digestible food components to beneficially modulate the gut microbes in the gastrointestinal tract (GIT) and thereby exerts health-promoting effects (Zheng et al., 2016). Due to non-digestibility in the stomach, these ingredients can be utilized by both bifidobacteria and lactobacilli in the lower GIT as a substrate during fermentation. Particularly, α-amylase enzymes are known to hydrolytically break the glycosidic bonds of the starch molecule and its derivatives (Li et al., 2014). In return, prebiotic manipulates the composition of colonic microbiota in GIT and ultimately improves the host health. Due to increasing demand for prebiotics, it is imperative to find novel sources of prebiotics which are relatively cost-effective. The studies on the validations of prebiotics are limited and only include commercial inulin, galactooligosaccharides (GOSs), fructooligosaccharides, and xylooligosaccharides (Belizario and Napolitano, 2015).

Previous studies claimed resistant starch can act as an effective ingredient to change the gut composition (Fuentes-Zaragoza et al., 2010). Similarly, potato (Zhang et al., 2013; Liang and McDonald, 2014; Liang et al., 2015; Zheng et al., 2016) and bamboo shoots (BSs; Azmi et al., 2012; He et al., 2016) have been studied for their prebiotic attributes due to the high percentage of polysaccharides which can act as substrates to enhance the colonic fermentation by increasing the number of *Bifidobacterium spp*. and *Lactobacillus spp*. Despite the available data for the prebiotic effects of potato and BSs, there is still scope to understand the suitability of potato peel (PP) and BS extracts as bacterial fermentation substrates. Due to the presence of sufficient amount of starch, cellulose, hemicelluloses, and fermentable sugars in PP (Jeddou et al., 2016), yeasts have been employed for exploiting PP for ethanol production and also *Bacillus* species have been studied for enzyme production. BS extract as a potential starting material to achieve the enhanced fermentation also offers an alternative strategy to reduce the series of steps involved in the purification and identification of polysaccharides.

Among the functional attributes, extracellular polysaccharide [exopolysaccharide (EPS)] production by LAB influences the physicochemical behavior of fermented foods and contributes toward “functional” foods by interacting with the immune system of consumers (Caggianiello et al., 2016), guards the bacterial cells against severe stress, and provides adhesion to surfaces and biofilm formation (Yuksekdag and Aslim, 2008).

In the recent times, use of indigenous LAB for fermentation process as functional starters has become increasingly necessary (Zeng et al., 2014). The native LAB derived from human feces or fermented foods represent ideal candidates for starter cultures. Moreover, the molds and yeasts lead to spoilage of various fermented dairy and vegetable foods which ultimately lead to a significant economic loss for food producers. Previously, several reports claimed the antifungal activities of the LAB and their possible applications as biopreservatives which resist the fungal growth during storage period (Tropcheva et al., 2014).

Since many years, microencapsulation stabilizes the probiotic cells and significantly enhances their stability by delivering the large number of viable strains of probiotic to consumers by overcoming the acidic environments (Gbassi et al., 2009; Chavarri et al., 2010; Khalil et al., 2015; Moumita et al., 2016). Bioprospecting better probiotics and making them more robust by encapsulation improve their endurance and enrichment with appropriate prebiotics can enhance the chance of successful delivery in a sufficient amount. The study reported herein promotes the ability of PP and BS extracts to enhance the bacterial growth and ensures the resistance to simulated GI stress conditions. Furthermore, the isolated LAB were evaluated for their *in vitro* functional attributes followed by microencapsulation of the selected LAB strain.

## Materials and Methods

### Raw Materials, Isolation, and Identification of LAB Strains

The BS and potatoes were procured from NingguoMaosheng Food Co., Ltd. (Anhui, China) and local market (Hefei, China), respectively. The PPs were dried at 60°C followed by fine grinding using a TQ-2000Y grinder (Rui-he Experimental Equipment, Zhejiang, China) and passing through a 60-mesh sieve, and then stored in polyethylene bags until use. The same procedure was followed for BS except drying. To obtain the BS and PP extracts, dried PP and BS materials (35 g) were added into 350 mL of absolute ethanol with 5% acetic acid (95:5 ratio) and allowed to stand for 72 h with the magnetic stirrer. Both the samples were vacuum-filtered by using Whatman No. 1 paper. The obtained residues were subjected to sonication for 20 min with 50 mL of the extraction solution at 20°C. The resulting extract was concentrated in a rotary evaporator (Shanghai Yarong Biochemistry Instrument Factory, China; Farvin et al., 2012). The fermented vegetables (7), fermented milk products (4), and human feces (12) were obtained from nearby supermarkets and Hefei University Technology (Hefei, China) volunteer students, respectively. As per our previous study (Thakur et al., 2015), the samples were processed for bacteria isolation by following the serial dilution (0.8% NaCl) through pour plating on De Man, Rogosa, and Sharpe (MRS) agar plates. The obtained colonies were subjected to phenotypic and genotypic characterization.

### Fermentation Medium and Analysis of BS and PP Prebiotic Activities

The fermentation media preparation was followed as per the method of Zhang et al. (2013). Briefly, all the fermentation experiments were conducted in a MRS medium containing three types of carbohydrates as glucose, PP, and BS extracts in the form of suspensions obtained by mixing with 0.5, 1.0, and 2.0% for 0, 12, 24, 36, and 48 h. After every interval, the OD_600_ nm and pH values were analyzed at 0, 12, 24, 36, and 48 h. The control sample consisted of LAB in a fermentation medium with well-known prebiotic, inulin. All the fermentation experiments were conducted in triplicates.

### *In vitro* Digestibility of PP and BS Extracts

The inability of human enzymes to digest oligosaccharides represents the ability to resist the gastric pH, breakdown by mammalian enzymes and absorption (Wang, 2009). The degree of hydrolysis was calculated when subjected to artificial human gastrointestinal juice (Chavarri et al., 2010; Brinques and Ayub, 2011) as per the previously described method (Azmi et al., 2012). The sample solutions (1% w/v, 5 mL) were exposed to simulated gastric liquid (5 mL at 1, 2, 3, and 3.5 pH) and intestinal juice (5 mL at pH 7, 8, and 9). The obtained sample solution (1 mL) was removed at specific time intervals such as 1, 2, 3, 4, 5, and 6 h for determination of reducing sugar (Robertson et al., 2001) and also total sugar content (Dubois et al., 1956). In this experiment, inulin was used as a positive control.

### Amylase Activity

In order to confirm the utilization of starch content, LAB were examined for amylase production as per previous methods (Sanni et al., 2002; Owusu-Kwarteng et al., 2015). Briefly, log phase cells were spread on MRS agar supplemented with 2 mg/L PP starch and incubated at 37°C for 48 h. To observe the starch hydrolysis, the incubated plates were taken out and covered with Lugol’s iodine [0.33% (w/v) iodine, 0.66% (w/v) potassium iodide]. The appearance of blue-black zone indicated the un-degraded starch, while the clear halo zone showed the starch degradation by α-amylase.

### Exopolysaccharide Production

The influence of different concentrations of carbons (glucose, sucrose, and fructose) on EPS production and growth of LAB was studied (Yuksekdag and Aslim, 2008). Active cultures were streaked on MRS agar containing different concentrations of carbon sources and at different pH to obtain the maximum EPS production after 48 h. The slimy colonies tentatively considered positive for EPS production were further confirmed using MRS–sucrose broth. The confirmed isolates were incubated at 30°C for 24 h followed by centrifugation at 5000 *g* for 10 min at 4°C. Then after, 1 mL of the resultant supernatant was transferred into an equal volume of ethanol (99%). In the presence of EPS, an opaque ring was expected to appear at the interface. For EPS production, phenol-sulphuric method (Dubois et al., 1956) was used and glucose was taken as a standard (Torino et al., 2001).

### *In vitro* Probiotic Tests

The acid tolerance and cell surface hydrophobicity (xylene and chloroform) were tested as per our previous study (Thakur and Tomar, 2016) at 0, 30, 60, 90, and 120 min at pH 2. For aggregation property, freshly grown LAB were centrifuged and resuspended in 4 mL of phosphate buffer saline (PBS) and the initial absorbance was read at 600 nm followed by incubation for 3 h at 37°C. At a regular interval of 1 h, from the top of the upper layer, 100 μL was mixed with 3.9 mL of PBS and the absorbance was read at 600 nm.

### Microencapsulation of Selected Strain and Survival of LAB Under *in vitro* Simulated Gastric and Intestinal Juices

Microencapsulation method was followed as per the previous report by Zanjani et al. (2014). Two grams of potato starch was dissolved into 100 mL of distilled water and kept for boiling until the formation of a gel, followed by addition of sodium alginate and inulin (1%). The selected LAB were added to the above solutions and stirred continuously for uniform distribution of the bacteria followed by transfer into 500 mL of vegetable oil supplemented with 0.2% tween 80. The whole solution was vigorously mixed until its creamy appearance. Subsequently, 200 mL of calcium chloride solution (0.1 M) was added into the mixture for phase separation. The calcium alginate capsules were allowed to settle at the bottom of the beaker followed by removing the oil layer. Then after, the retained capsules were harvested by low-speed centrifuge at 350 g for 10 min and kept in 0.1% peptone solution at 4°C until next use. Simulated gastric juice with pepsin (0.5%) and simulated intestinal juice with pancreatin (0.5%) were filtered through a 0.22 μm membrane. The selected (encapsulated and non-capsulated) LAB exposed to sterile juices were incubated at 37°C for 30, 60, 90, and 120 min. Surviving bacteria were calculated by plating on MRS agar and % survivability was calculated to evaluate the protective effect of encapsulation.

### Technological Attributes

To determine the starter activity, 20 mL of heat treated (90°C for 10 min) 11.5% reconstituted skimmed milk was inoculated with active cultures (2%) and kept at 37°C (Thakur et al., 2016a). For acid production, active culture (1%) were inoculated and kept at 37°C for 6 and 24 h for measuring the percentage of lactic acid produced. For proteolytic activity, the identified isolates were cultivated on reconstructed agar plates containing 10% of skim milk medium followed by incubation at 30°C for 18–20 h. Colonies having a transparent zone around them indicated the proteolytic activity (Swearingen et al., 2001). Further, the antifungal activity of test cultures was checked in MRS plates using the overlay technique. Test cultures were spread on the MRS Petri plates and incubated at 30°C for 18 h. After their active growth, 10 mL of spores (bread mold) were added and incubated at 25°C. Thereafter, the growth of plated mold and inhibition clear zone in the areas of growth of test LAB were evaluated.

### Statistical Analysis

One-way analysis of variance (ANOVA) using Origin Lab (Origin Pro 8.0) software was used for data analysis at a significance level of *p* < 0.05. All the data were expressed as mean ± SD (*n* ≥ 3). Significant differences between the experimental conditions were evaluated by Duncan’s multiple range test at *p* < 0.05.

## Results

### Identification of LAB and Phylogenetic Tree Construction

**FIGURE 1:**
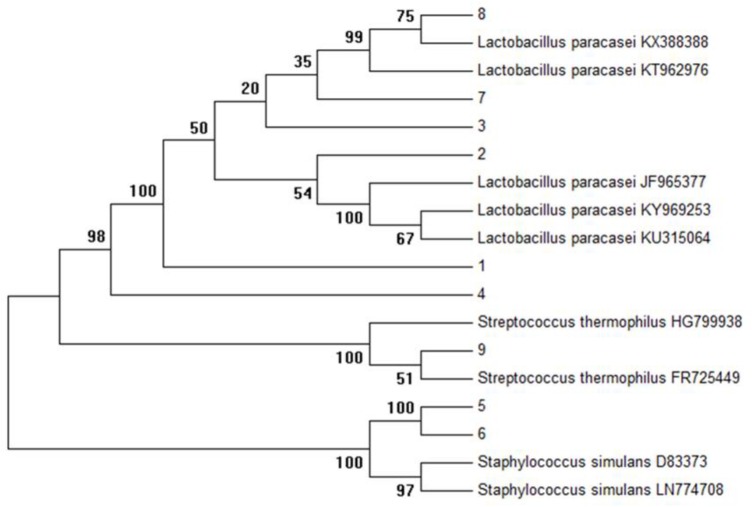
Phylogenetic tree of all isolates (K1–K9) was constructed using the UPGMA method by Mega 6.0 analysis (16S r DNA sequences of reference strains were obtained from the NCBI database).

Initially, on the basis of gram staining (rod-shaped), 32 isolates were characterized by 16s primers with a product of expected size (1500 bp). As shown in **Figure 1**, the complete sequencing of 16S rDNA and phylogenetic analysis revealed the selected isolates as *Lactobacillus paracasei* (6), *Staphylococcus simulans* (2), and *Streptococcus thermophilus* (1). The complete 16s sequences were submitted to National Center for Biotechnology Information Gene bank.

### Proliferation of LAB (Growth Curve, Change in pH, and Acidity) in the Presence of PP and BS Extracts

**FIGURE 2:**
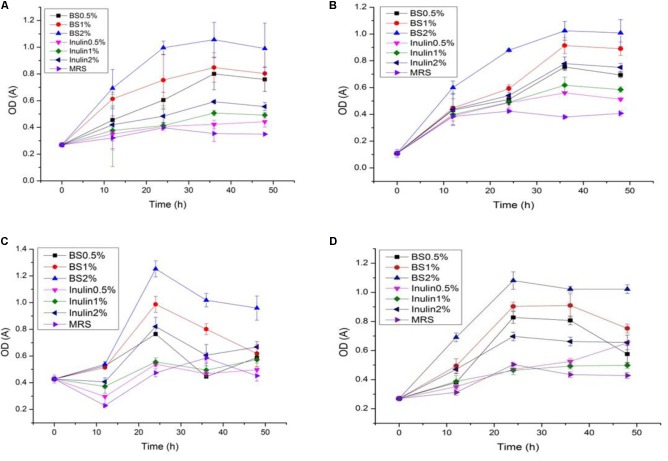
**(A–D)** The cell density change of selected LAB (1, 3, 4, and 9) strains in the presence of different concentrations of BS (0.5, 1, and 2%) over fermentation time (at 600 nm).

**FIGURE 3:**
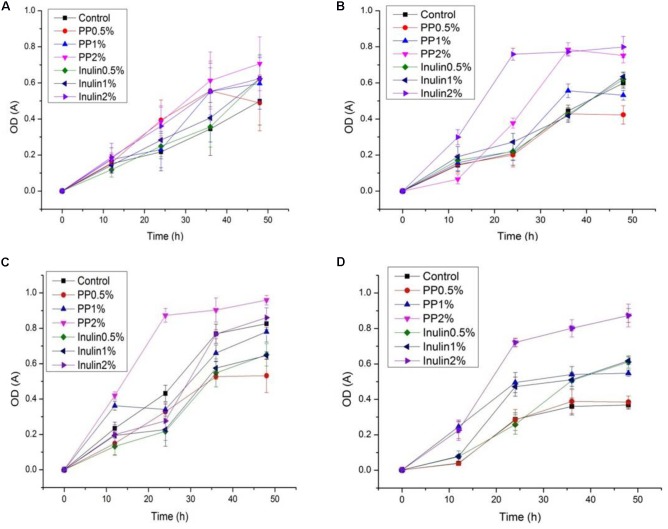
**(A–D)** The cell density change of selected LAB (1, 3, 4, and 9) strains in the presence of different concentrations of PP (0.5, 1, and 2%) over fermentation time (at 600 nm).

**FIGURE 4:**
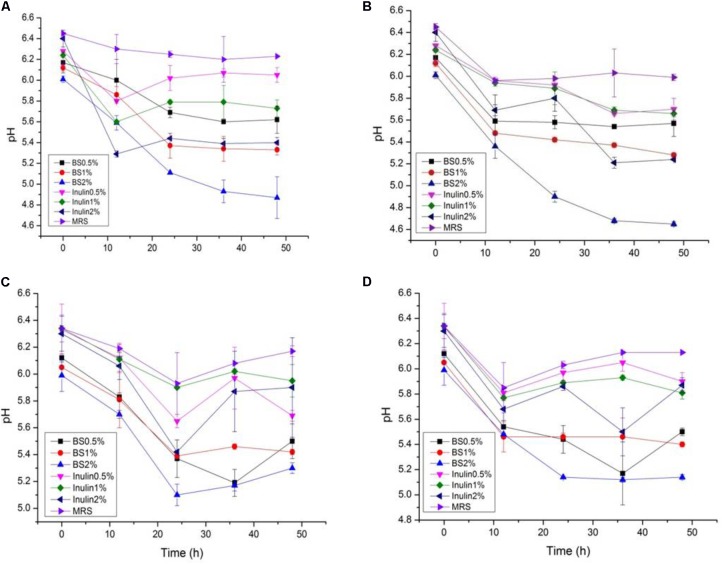
**(A–D)** The pH change of selected LAB (1, 3, 4, and 9) strains in the presence of different concentrations of BS (0.5, 1, and 2%) over fermentation time (at 600 nm).

**FIGURE 5:**
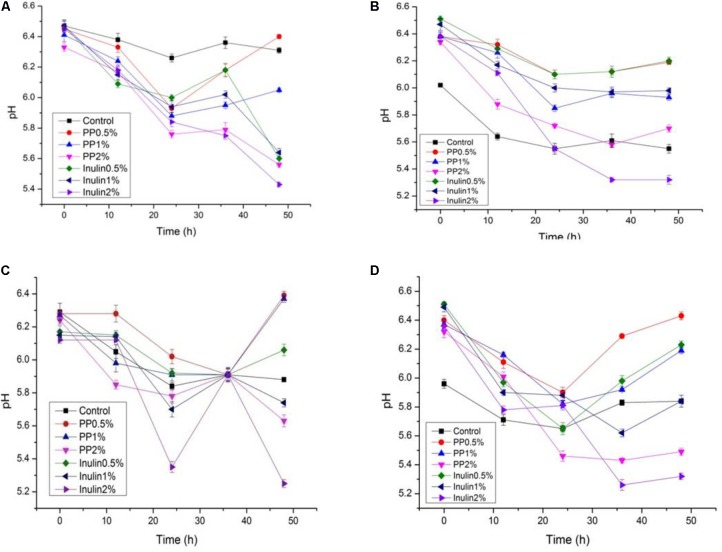
**(A–D)** The pH change of selected LAB (1, 3, 4, and 9) strains in the presence of different concentrations of PP (0.5, 1, and 2%) over fermentation time (at 600 nm).

The LAB proliferation in the media with different carbon sources was presented in **Figures 2**–**5**. Compared with the media containing glucose or inulin, significantly higher (*p* < 0.05) OD_600_ nm values appeared in the media containing 2% BS and PP (**Figures 2**, **3**). As per OD_600_ nm value, the density of LAB supplemented with BS was observed at the highest levels after different intervals of fermentation. The increasing concentration of carbon sources led to a rapid reduction in pH values (**Figures 4**, **5**) over the period of fermentation. For the prebiotic activity test, the color of fermented broth turned to turbid deep yellow after incubation, indicating fermentation of added carbon substrates. In particular, BS (2%) have significantly increased the growth of LAB comparable to inulin and PP. All the tested LAB showed an appreciable rise in growth density of about 0.2–1.2 as well as rapid pH change after 12 h was noticed by using BS rather than inulin and PP. The viability of tested LAB in BS and PP supplemented media potentially displayed a positive increase up to 36 h for K1 and K4 strains.

### *In vitro* Tolerance Toward Simulated GIT Conditions

**FIGURE 6:**
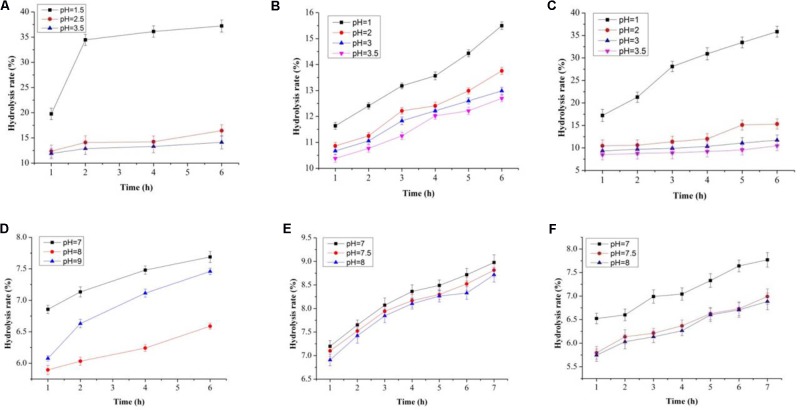
Degree of hydrolysis in artificial human gastric juice for BS **(A)**, PP **(B)**, inulin **(C)**, and degree of hydrolysis in human intestinal juice for BS **(D)**, PP **(E)**, and inulin **(F)**.

**FIGURE 7:**
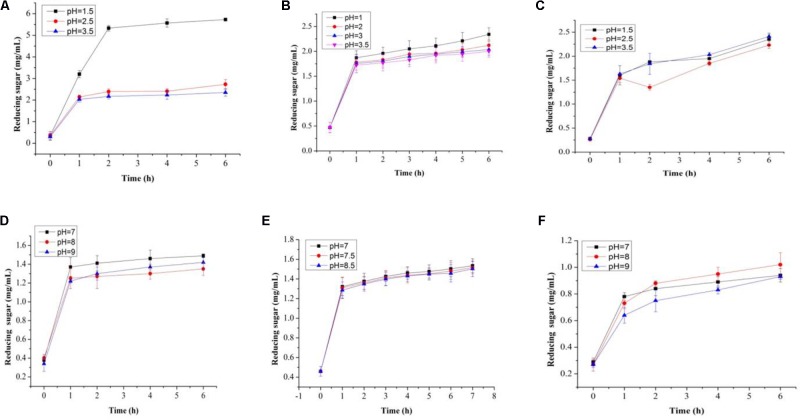
Total reducing sugars (mg/mL) in artificial human gastric juice for BS **(A)**, PP **(B)**, inulin **(C)**, and total reducing sugars (mg/mL) in human intestinal juice for BS **(D)**, PP **(E)**, and inulin **(F)**.

In this study, both the tested samples (PP and BS) were observed to be non-digestible when exposed to artificial human gastrointestinal juice as compared to inulin (**Figures 6**, **7**). Interestingly, the non-digestibility rate of BS and PP was very high at the pH tested (pH 2.5) with the differences in the degree of hydrolysis (0.12 ± 0.06 to 0.23 ± 0.09%) from 0 to 6 h at all the pH tested as compared to inulin which ranged from 0.01 ± 0.09 to 0.07 ± 0.03% (**Figure 6**). The present data revealed the moderate susceptibility of BS for gastrointestinal juice at all the time intervals and pH tested. Moreover, after the uptake of food by our body, it is retained in the gastric environment for about 2 h (Wang, 2009). Therefore, our results could support the significant use of BS and PP as a potential prebiotic due to their non-digestibility which help them to reach the GIT completely intact and readily available for modulating gut microbes.

### *In vitro* Probiotic Properties

**FIGURE 8:**
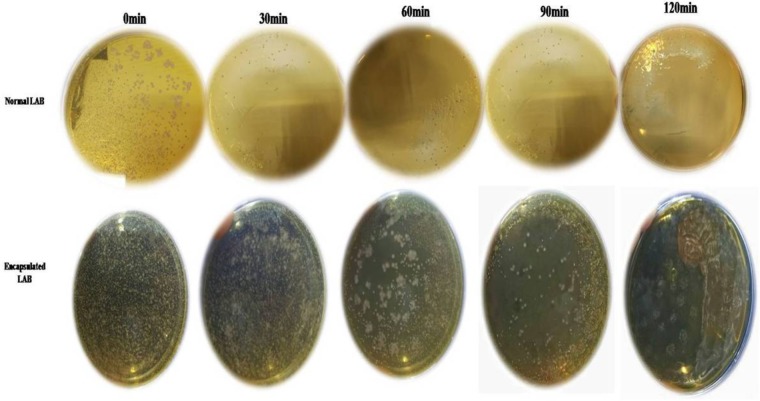
Survival rate of normal LAB and encapsulated LAB exposed to pH 2.0 after 0, 30, 60, 90, and 120 h intervals.

**FIGURE 9:**
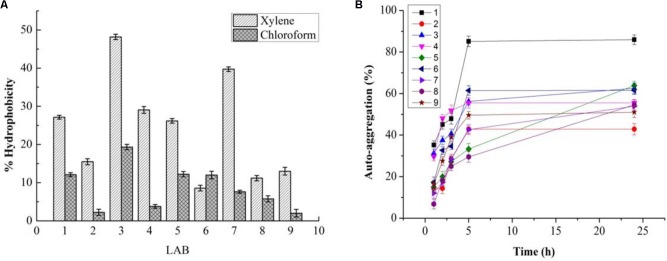
Cell surface hydrophobicity **(A)** and % autoaggregation **(B)** in LAB (1, 2, 3, 4, 7, 8, and 9) and *two Staphylococcus simulans* strains (5 and 6).

While PP and BS have valuable effects on the growth of LAB, the latter can be affected by GIT conditions. Therefore, the effects of PP, BS, and inulin on the tolerance ability of the LAB to gastrointestinal conditions were assessed. There was a significant decrease in the survival rate of the LAB in media (pH 2.0) with PP, BS, and inulin following 120 min of incubation. When the pH values of the medium were 2.0, the survival rate of the LAB decreased after 90 and 120 min of incubation. At low pH values, the LAB in the BS or PP supplemented media have shown better adaption to low pH values than in glucose medium. Our data revealed that 50% of LAB survived in the BS medium after 30 min of incubation. On the other hand, no LAB were detected in the control MRS media after 60 min. Therefore, BS increased the resistance of LAB to low pH values. This effect was further enhanced by increased survival of an encapsulated LAB (K3) under simulated GIT. The significant increase in the survival rate of the encapsulated LAB was observed after 120 min under simulated GIT conditions as compared to the free LAB (**Figure 8**). The survival rate after 120 min of incubation was more than 70% for the encapsulated LAB. The strains (K3 and K7) and strains (K1and K6) showed appreciable cell surface hydrophobicity (**Figure 9A**) and autoaggregation (**Figure 9B**) properties, respectively.

### Exopolysaccharide Production

**FIGURE 10:**
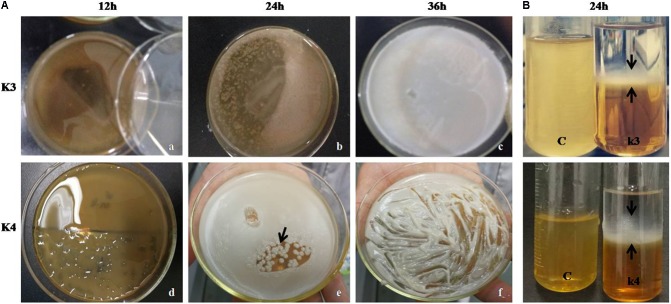
**(A)** LAB colonies (K3 showing shiny flat mat) and K4 with shiny convex colonies on MRS-sucrose agar medium at 12 **(a,d)**, 24 **(b,e)**, and 36 h **(c,f)**. **(B)** EPS precipitation by ethanol showing opaque ring link in the interface (black arrows) in two LAB strains and **C** represents blank MRS medium.

**FIGURE 11:**
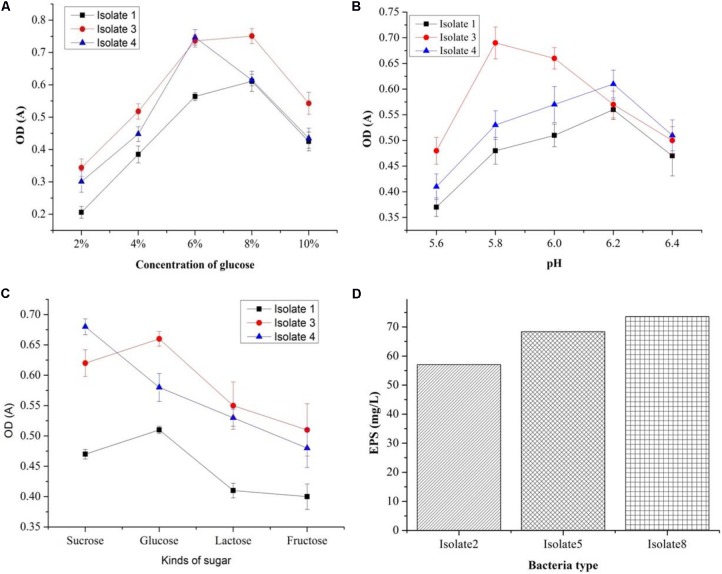
Optimization of EPS production conditions: effect of different glucose concentrations **(A)**, effect of sugar types **(B)**, effect of sugar type on cell density **(C)**, and EPS production (mg/L) by three bacteria isolates **(D)**.

Out of seven strains of the LAB, only three showed slime formation (**Figure 10A**) and an opaque ring was observed at the interface of test tubes (**Figure 10B**), while remaining LAB showed no slime formation or EPS production. The positive significant link between the EPS production at the maximum carbon content (*p* < 0.05) was noticed. For the strains, K1, K4, and K3, the optimal glucose and sucrose concentrations were 8, 8, and 7 g/L at pH 6.3, 5.9, and 6.2 (**Figures 11A–C**) for the maximum EPS production of 57.03 ± 0.09, 68.35 ± 0.023, and 73.58 ± 0.011 mg/L, respectively (**Figure 11D**).

### Technological Attributes

#### Change in pH and Acidity

**FIGURE 12:**
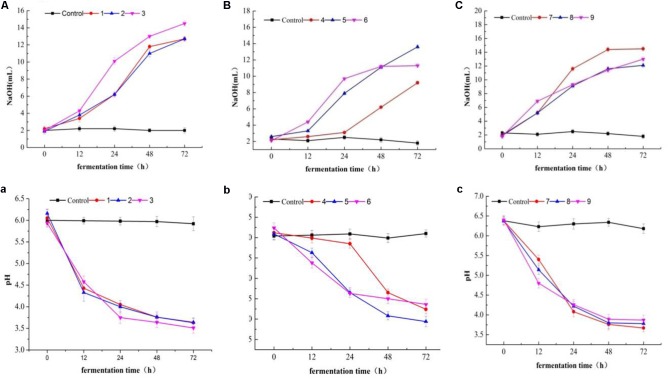
Acidity **(A–C)** and pH **(a–c)** changes in milk over fermentation time (0, 12, 24, 48, and 74 h) in LAB strains (1, 2, 3, 4, 7, 8, and 9) and two *Staphylococcus simulans* strains (5 and 6).

Lactic acid bacteria were grouped into two categories due to their acidification (**Figures 12A–C)** and pH change performance (**Figures 12a–c**) in milk. Most of the tested isolates were characterized as fast acidifiers, whereas K1 showed the maximum acidity change as compared to the remaining isolates (**Figure 12A**). The demonstration of faster acidification indicates the better fermentation processes and reduces the fermentation time and poses less risk of contamination by spoilage and/ or pathogenic microorganisms.

#### Proteolytic, Lipolytic, and α-Amylase Activity

**FIGURE 13:**
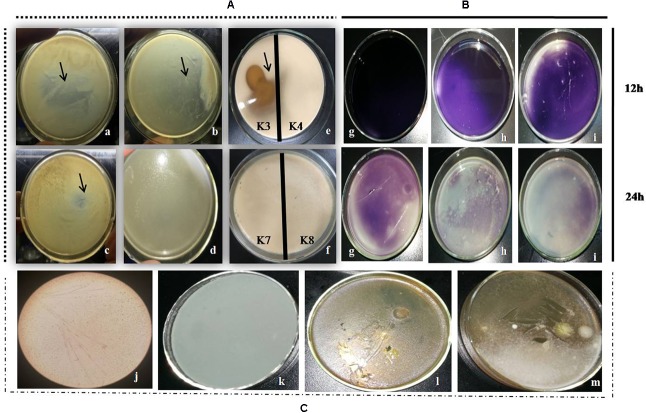
**(A, a–d)** Transparent zone for proteolytic activity of K3, K4, K7, and K9(–ve), **(e)** the lipolytic activity of K3 and K4(–ve), and **(f)** no lipolytic activity by K7 and K9. **(B)** The amylase activity for starch hydrolysis by K1 **(g)**, K3 **(h)**, and K4 **(i)** after 12 and 24 h. **(C)** The antifungal action against bread mold **(j)** and **(k)** representing the control without LAB and K3 **(l)** and K4 **(m)** representing test strains after 5 days of incubation.

In the present study, only three LAB strains, namely, K3, K4, and K8 have shown the considerable hydrolysis of milk proteins (transparent zone)as shown in **Figures 13A(a–d)** as well as the moderate lipolytic activity by showing clear zone in the center of the Petri plates [**Figures 13A(e,f)**]. Amylase activity of LAB was shown in **Figure 13B(g–i)**. The α-amylase activity of the three strains [**Figure 13A(g–i)**] was observed to be appreciable except three strains which only showed weak activity or no detection.

#### Antifungal Action

The substantial antifungal activity of LAB strains against the bread mold was observed for selected strains. Among the tested strains, only two strains could resist the fungal growth by the end of the experiment; whereas, the entire surface of the Petri plate (green layer on the surface) was filled with mold growth [**Figures 13C(j–m)**]. The complete inhibition by strain K3, while moderate antifungal action of K4 strain [**Figure 13C(l)**] was observed.

## Discussion

In the present study, total seven LAB and two *Staphylococcus simulan* were identified. The species *S. simulans* was isolated and identified from the fermented vegetable source during the isolation of LAB. We have included this particular strain, not to claim its use as a starter culture but only to compare its activity with LAB. All isolates have shown a positive correlation toward the supplementation of PP and BS extracts. As evident from the previous study, due to the complex nature of BS extract, a longer time may be required for the bacteria to digest the polymer (Vardakou et al., 2008). After 72 h of fermentation, we anticipate the most of carbon sources were utilized and may not be sufficient for further use. Previous reports suggested the prebiotic activity of glucans extracted from *Pleurotus ostreatus, Pleurotus eryngii* by Synytsya et al. (2009), and fermented cashew apple (*Anacardium occidentale* L.) juice by Vergara et al. (2010). The decrease in pH values after incubation with BS and PP extracts suggested that LAB were able to utilize them. Interestingly, BS, PP, and inulin could support the growth of *Staphylococcus* spp.; however, the rise in cell density was minimal as compared to the LAB.

Bamboo shoot showed better tolerance to artificial human gastric juice as compared to inulin. The previous studies suggested 100, 98.4, and more than 90% resistant to artificial gastric acid by other oligosaccharides studied such as kojioligosaccharides (Nakada et al., 2003) and gluco-oligosaccharide (Wichienchot et al., 2006) and commercial prebiotic, a GOS (Beards et al., 2010), respectively. The higher resistance toward non-digestibility indicated that BS extract could reach the colon intact without any content loss. In the previous study, galactose-rich oligosaccharides/oligomers (81.6%) and its corresponding oligomers (79.3%) remained unhydrolyzed, respectively (Khodaei et al., 2016). PP has the potential to be used as prebiotic due to high starch content, but due to its low fermentable reducing sugar content, it might be appropriate to achieve the initial hydrolysis of carbohydrates(Zheng et al., 2016). Usually, amylase producing LAB are not very common except few strains of *L. fermentum* isolated from fermented maize products (Sanni et al., 2002). Due to the immense importance of LAB in fermentation industries, the developed fermented foods are affected by lactic acid production and decreased pH consequently (Zhang et al., 2013). Thus, in the search of new starter cultures, we could select potent LAB which can be used for desired fermentation in the future studies. The present study revealed that increased EPS production with rising glucose concentration. Yuksekdag and Aslim (2008) reported that carbon source and its concentration significantly affect the EPS production, whereas glucose was considered as the most efficient carbon source. Moreover, the composition of the medium (carbon and nitrogen sources), as well as incubation conditions such as temperature, pH, time, etc., also affect the EPS production (Yuksekdag and Aslim, 2008). Therefore, finding the new EPS-producing LAB with potential industrial relevance can be of interest by applying different optimized conditions (Looijesteijn et al., 2000). Moreover, prebiotic effects have also been observed for EPS produced by LAB as reported in the previous studies (Tsuda and Miyamoto, 2010; Das et al., 2014). Putative probiotics are often evaluated on the basis of acid and bile tolerance, cell surface hydrophobicity, and autoaggregation under *in vitro* conditions (Monteagudo-Mera et al., 2012; Xing et al., 2017). The ability to adhere to epithelial cells plays an important role as an essential criterion for probiotics selection (Kos et al., 2003). Previously reported, the aggregation ability is related to cell adherence properties. Therefore, we have examined the LAB and *Staphylococcus* sp. for their cell surface hydrophobicity and autoaggregation ability as probiotic screening criteria which indicate their binding capacity (Thakur and Tomar, 2016). Thus, high hydrophobicity and autoaggregation values of bacteria cells could lead to greater attractive forces and ultimately higher levels of adhesion. The approach for providing bacterial cells with a physical barrier against adverse environmental conditions has been receiving huge interest (Chavarri et al., 2010; Zanjani et al., 2014; Khalil et al., 2015). This study has demonstrated that the encapsulation of LAB which improved their survival under conditions simulating the human GIT and the resistance of bacteria to these conditions. Indeed, the reduction in the count of encapsulated cells was observed in an acidic solution containing pepsin after 60 min of incubation, while the free cells could not survive.

## Conclusion

Along with the previous reports, our results have added new insights in the existing knowledge for the use of plant-derived prebiotics and their protective roles which ensure an increased survival of bacterial strains to GIT stresses. This simple and novel approach can potentially decrease the cost of fermentation process besides maximizing the value of naturally abundant resources to produce several byproducts during desired fermentation conditions. The selected LAB with better functional attributes hold the promise to be used as starter cultures in the food and dairy industries. Taken together, current finding presents promising results on the potential use of PP and BS extracts as fermentation substrates which not only reduces the cost of carbon substrates but also alleviates the desired fermentation goals by converting environmental waste into suitable biomass. However, the interaction between the LAB and natural extracts is a complex phenomenon since there is simultaneous competition for degradation products. Thus, future studies are warranted to explore the use of purified individual components and their individual effects on gut modulation in suitable mice models.

## Author Contributions

KT designed the work and G-YX executed the experiments and analysis. KT prepared the manuscript draft and revised the manuscript. J-GZ carried out the interpretation of data, drawing up figures and statistical analysis. KT and G-YX carried out the microbial growth analysis, *in vitro* tests, and microencapsulation experiments. FH and FZ helped in data analysis. Z-JW contributed to the experimental design, manuscript preparation, and submission. All authors read and approved the final manuscript.

## Conflict of Interest Statement

The authors declare that the research was conducted in the absence of any commercial or financial relationships that could be construed as a potential conflict of interest.
